# The Evolving Protein Engineering in the Design of Chimeric Antigen Receptor T Cells

**DOI:** 10.3390/ijms21010204

**Published:** 2019-12-27

**Authors:** Hannah E. Hughes-Parry, Ryan S. Cross, Misty R. Jenkins

**Affiliations:** 1 Immunology Division, The Walter and Eliza Hall Institute of Medical Research, Parkville, VIC 3052, Australia; Hughes-Parry.h@wehi.edu.au (H.E.H.-P.); cross.r@wehi.edu.au (R.S.C.); 2 Department of Medical Biology, The University of Melbourne, Parkville, VIC 3052, Australia; 3 Institute of Molecular Science, La Trobe University, Bundoora, VIC 3086, Australia

**Keywords:** immunotherapy, chimeric antigen receptor T cells (CAR T cells), affinity tuning, dual chain CAR T cells (dcCAR), ligand-based CAR T cells, T cell receptor fusion constructs (TRuCs), universal immune receptors (UIR), dual CAR T cells, tandem CARs (tanCARs), bispecific T cell engagers (BiTEs)

## Abstract

The clinical success of chimeric antigen receptor (CAR) T cell immunotherapy in the treatment of haematological cancers has encouraged the extensive development of CAR design to improve their function and increase their applicability. Advancements in protein engineering have seen modifications to both the ecto- and endo-domains of the CAR, with recent designs targeting multiple antigens and including inducible elements. These developments are likely to play an important role in inducing effective CAR T cell responses in a solid tumour context, where clinical responses have not been effective to date. This review highlights the spectrum of novel strategies being employed in CAR design, including for example variations in targeting tumour antigens by utilising different ectodomain designs such as dual chain CARs, natural receptor or ligand-based CARs, and T cell receptor fusion constructs, and also reviews some of the innovative approaches to a “universal” CAR and various multi-antigen targeting CAR strategies. We also explore how choices in the endodomain impact CAR function and how these need to be considered in the overall CAR design.

## 1. Introduction

**Figure 1:**
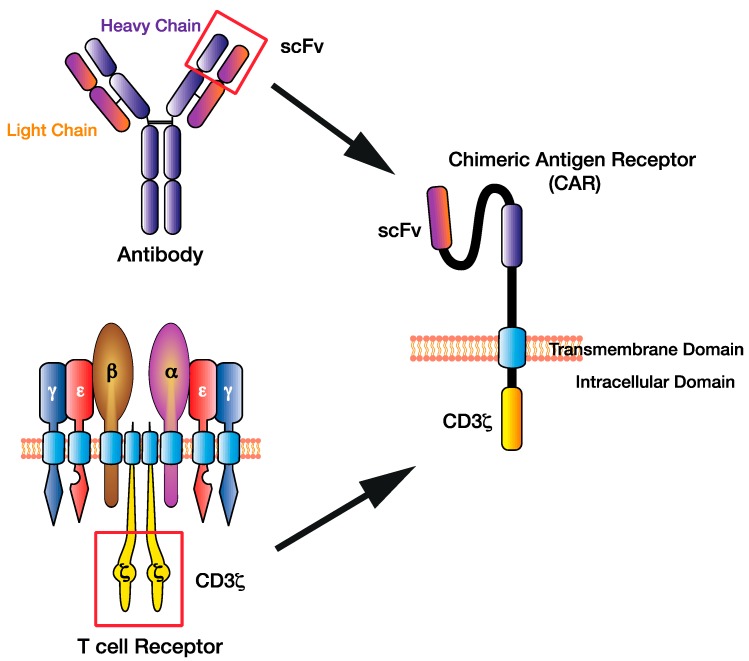
The chimeric antigen receptor (CAR) T cell design has evolved by combining existing immune cell components to facilitate direct targeting of tumour antigens. The single-chain variable fragment (scFv) of the CAR derived from the heavy and light chains of the antibody variable region, while the CAR CD3ζ domain is derived from the T cell receptor intracellular signalling domains.

Chimeric antigen receptors (CARs) are synthetic proteins engineered to be expressed on the cell surface of cytotoxic immune cells, such as T cells, to facilitate the enhanced recognition and elimination of malignant cells. A CAR consists of an antigen-binding ectodomain, a spacer linked to the transmembrane domain, and an endodomain commonly consisting of a costimulation domain and cluster of differentiation 3 (CD3) ζ signalling tail (Figure 1). Triggering of the ectodomain induces signalling via the CD3ζ endodomain (a critical component of the T cell receptor (TCR) facilitating signal transduction and exocytosis of cytotoxic granules) and apoptosis of the antigen-expressing cancer cell. The approach was first pioneered in the 1980s by Gross and colleagues, and involved the genetic engineering and ex vivo expansion of syngeneic T cells designed to directly target the patient tumour antigen [1].

T cell redirection strategies have become a novel advancement over historical approaches using adoptive T cell transfer [2,3], providing the advantage of allowing (1) major histocompatibility complex (MHC)-independent recognition of malignant cells through direct target antigen specificity, and (2) expansion of a large number of polyclonal T cells, all of which can be redirected to target malignant cells. The clinical efficacy of CD19 targeted CAR T cells led to two US Food and Drug Administration (FDA)-approvals in 2017, Kymriah in acute B cell lymphoblastic leukaemia (B-ALL) and Yescarta in diffuse large B-cell lymphoma (DLBCL) [4,5].

CAR design has evolved in terms of sophistication, with exquisite flexibility and controllability leading to applications beyond cancer [6,7]. To overcome early efficiency challenges, a single-chain antibody ectodomain was generated consisting of a single-chain variable fragment (scFv) from the heavy and light antibody variable regions [8] (Figure 1). This ectodomain transformed the chimeric receptor design, as it allowed a targeted approach of using antibodies to target cell surface antigens, including proteins, carbohydrates, or glycolipids, expanding the scope beyond TCR-restricted peptide–MHC targets.

**Figure 2:**
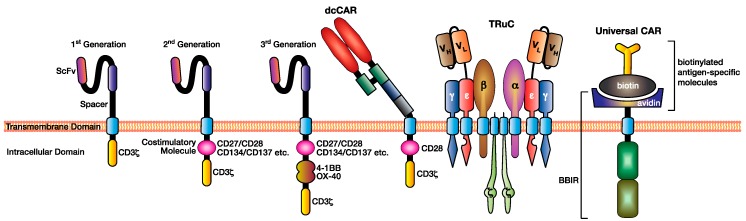
Various modifications have been made to the CAR design to facilitate superior antigen targeting, CAR T cell function, and applicability. This figure illustrates three generations of CAR design (first, second, and third) depending on the addition of costimulation domains, the dual chain CAR (dcCAR), the T cell receptor fusion construct (TRuC), and an example of a universal CAR utilising the biotin–avidin system.

The ectodomain is linked, using various transmembrane domains, to the gamma chain of the immunoglobulin receptor or the CD3ζ chain, which is sufficient to induce T cell activation in a tumour-antigen specific manner [8]. However, this “first-generation” CAR resulted in a lack of durable responses (Figure 2). The addition of a CD28 costimulation domain to create “second-generation” CARs targeting CD19 resulted in increased CAR T-cell persistence in vivo and in vitro [9]. Subsequent studies have highlighted the importance and flexibility of tailoring various domains of the CAR to formulate an optimal CAR T cell response. For example, the inclusion of two costimulation domains (“third” generation CAR) or even three (“fourth” generation) has shown to increase T cell activation, proliferation, and persistence, though the optimal combination of costimulatory domains is yet to be determined and is likely target- and tumour-dependent [10,11]. However, it is clear that the customisation of this “plug-and-play” approach can be used to optimise T cell function and tumour-targeting depending on the desired output.

In this review, we discuss the approaches currently used to fine-tune CARs to modulate T cell specificity and function. We will also summarise the recent developments in protein engineering which are also being applied to improve function and safety.

## 2. Designing the CAR: Selecting the Antigen Recognition Domain

An scFv-based ectodomain is the most common antigen-binding motif used in CARs, due to its ease of appropriation from existing antigen-specific monoclonal antibodies [12]. The incorporation of scFv binding domains has enabled antigen recognition with a range of affinities, but typically in orders of magnitude higher than TCR–peptide MHC (pMHC) interactions. In recent years, significant knowledge has been generated in the field regarding the effect of antibody modifications to the scFv ectodomain on CAR T cell efficacy and function, such as by tuning specificity or re-engineering CAR structures to other binding domains such as dual chains, native receptors, or ligand-based designs.

### 2.1. Affinity Tuning Antibody Domains

Single-chain antibody domains are amenable to affinity tuning, whereby the binding affinity between antigen and antigen-binding ectodomain can be selectively mutated, influencing affinity and CAR T cell function. Whilst CAR T research has traditionally utilised high affinity scFvs, there is some evidence to suggest that targeting the antigen using the highest affinity scFv possible does not necessarily result in the optimal outcome. Early studies using human epidermal growth factor receptor 2 (HER2)–CAR T cells with varying affinities (3.2 × 10^−7^ to 1.5 × 10^−11^ K_d_) has demonstrated that the activation threshold of CAR T cells is inversely correlated with scFv affinity [13]. More recently, evidence has suggested that high affinity TCR interactions (above 10 µM) increase T cell signalling strength, as determined by T cell calcium flux and extracellular signal-regulated kinase (ERK) phosphorylation, without necessarily improving the efficacy of T cell therapy in B16/A2-K^b^ melanoma mouse models [14]. These results suggest that high affinity binding domains may not necessarily improve therapeutic outcome. Affinity of binding has been shown to be critical in a clinical trial using trastuzumab (Herceptin), a widely used high affinity monoclonal antibody to HER2. In this trial of metastatic colon cancer, the high affinity HER2-specific CAR T cells also recognised low-level HER2 antigen expression on healthy lung epithelial cells, causing a cytokine storm and resulting in the death of a patient [15]. Conversely, in other phase I clinical trials, targeting HER2 with a lower affinity scFv has been shown to be safe [16]. This discrepancy highlights that the affinity of the scFv can selectively detect varying antigen loads and drive on-target off-tumour T cell responses. Researchers demonstrated that the selectivity threshold to antigen could be controlled, as tuning trastuzumab-based CARs to a lower affinity could spare “healthy” tissue levels of HER2 on PC3 cells whilst maintaining efficacy against “high” tumour levels of antigen expression on HER2-overexpressing SK-OV3 cells [17]. Therefore, controlling the affinity and avidity of CAR T cell interactions is of critical importance when designing a CAR targeting tumour-associated antigens (TAAs) which may also be expressed on healthy tissues. These findings have been replicated in other studies involving the affinity tuning of scFvs against TAAs such as epidermal growth factor receptor (EGFR), CD123, and CD38 [17,18,19,20].

### 2.2. Dual Chain CAR (dcCAR)

A dual chain CAR-based approach is to use the antibody binding domain in its natural form as a heterodimer. In this format, both an immunoglobulin light and heavy chain are expressed simultaneously, linked via endogenous disulphide bonds with their constant region and fused to signalling endodomains [21] (Figure 2). This CAR format was shown to form stable heterodimers in in vitro cell lines, providing some evidence for enhanced dual chain CAR stability over the scFv format, facilitating equivalent cytotoxicity but lower levels of cytokine secretion compared to its scFv CAR equivalent [21]. The benefit of this approach is that, in theory, all monoclonal antibodies could be used in this format as some scFvs, such as a GD2.28z CAR, can cause antigen-independent clustering and tonic CAR signalling, leading to early CAR T cell exhaustion and reduced anti-tumour efficacy [22]. Whilst there is some evidence to suggest that the dual chain CAR design could result in lower cytokine production by the CAR T cells, which is desirable to prevent cytokine release syndrome (CRS), this has yet to be explored using a wider array of monoclonal antibodies to determine if it is a feature of dcCARs or of this particular clone [15,23,24,25].

### 2.3. T Cell Receptor Fusion Constructs (TRuCs)

It has been hypothesised that the additional costimulation signalling received by T cells in the periphery contributes to T cell exhaustion and lack of persistence [26]. To overcome this challenge, T cell receptor fusion constructs (TRuCs) were generated whereby an scFv ectodomain is incorporated into the endogenous human TCR via tethering to various CD3 subunits, providing antibody-based antigen recognition while maintaining T cell signalling via the entire TCR complex [27] (Figure 2). Baeuerle and colleagues demonstrated that TRuCs and CARs, targeting CD19 with identical scFvs, displayed similar in vitro killing kinetics and cytokine production [27]. However, TRuCs were shown to be superior in vivo in the clearance of both haematological and solid cancer xenograft mouse models, using Nalm-6 leukaemia, Raji lymphoma, and U251 glioblastoma cell lines [27]. However, whether the TRuC or CAR T cells are more effective in the clinic remains to be examined. Hofmeister and colleagues propose that in vivo improvements observed in the TRuC T cell response (such as increased tumour elimination with fewer TRuC T cells) compared to the equivalent CAR T cell response are due to signalling via the endogenous TCR [27].

### 2.4. Receptor and Ligand-Based CAR

Relatively less is known about the clinical efficacy of the natural ligand-based CAR, which involves the fusion of a membrane-tethered ligand (such as the interleukin (IL)-13 ligand recognising a tumour-associated or specific receptor such as IL13Rα2) to the CAR endodomains [28]. This approach is new, with few, such as the IL-13Rα2 and T1E CARs, having been translated to the clinic [28,29,30]. In one such promising trial, a single patient receiving multiple doses of CAR T cells targeting IL-13Rα2 demonstrated tumour regression of all intracranial and spinal recurrent multifocal glioblastoma in a phase I clinical trial; however, the patient relapsed 7.5 months following therapy [29].

Another ligand CAR is the T1E CAR (named after the promiscuous T1E peptide) targeting HER2/ErbB2 and ErbB dimers associated with many solid tumours, which entered a phase I clinical trial for squamous cell head and neck cancers (NCT01818323). This clinical trial was initiated following promising results using in vivo xenograft models where no accompanying toxicity was observed [30]. The T1E CAR was co-expressed in T cells alongside a chimeric cytokine receptor termed 4αβ, consisting of an IL-4 receptor a subunit ectodomain fused to the IL-2/IL-15 receptor β chain transmembrane and endodomain to facilitate gene-modified cell enrichment following IL-4 administration [31]. The use of the ErbB ligand allowed native binding to ErbB1- and ErbB4-based homo- and hetero-dimers, and ErbB2/3 heterodimers, which would not have been possible using any other CAR format. Other ligand-based CAR designs currently in pre-clinical testing include CARs targeting adnectin [32], follicle-stimulating hormone (FSH) [33], and CD27 (targeting CD70) [34]. The expansion of the CAR ectodomain to include designs beyond antibody CDR binding moieties has allowed a broader range of potential antigens to be targeted, particularly those with more complex binding interactions reliant on heterodimer/multimer receptors.

### 2.5. Universal Immune Receptors (UIRs)

Current approved CAR T cell immunotherapies are manufactured on a patient-specific basis, which limits agile responses to antigen escape and limits the ability for dose titration or repeat administration of CAR T cells. One approach to overcome these obstacles is to design an “off-the-shelf” flexible product. It is becoming clear that targeting a single cancer antigen may be insufficient for lasting remission. Even after early CAR T cell clinical successes, antigen escape, which is the persistence and outgrowth of antigen-negative tumour cells, remains a significant problem in the field, both for haematological cancers [35] and also solid cancers [36]. To mitigate antigen escape, recent CAR designs have adopted a “lock-key” system, relying on an intermediate molecule in antigen targeting to allow for broader antigen specificity. This approach results in the creation of a “universal” CAR which can then be used with various adaptor molecules.

There are several different kinds of UIRs with adaptable specificity, including (1) antibody dependent cytotoxicity receptors, (2) bispecific protein mediated linkage, (3) anti-Tag CARs, and (4) tag-specific interactions (reviewed extensively in [37]).

One form of UIR utilises the antibody dependent cytotoxicity receptors on effector cells, such as natural killer (NK) cells, to facilitate antibody dependent cell cytotoxicity (ADCC) of target tumour cells by incorporating receptors such as NKG2D [38], CD16 [39,40,41], NKp30 (targeting B7116, BAG6, Gal3) [42], or DNAM-1 (targeting PVR and adnectin) [43] as the antigen binding domains of the CAR. In particular, T cells transduced with CD16 CARs, which consists of an extracellular CD16 (or FcyRIIIa) domain attached to an FceRIy endodomain, cause ADCC or the antibody-mediated apoptosis of target tumour cells coated with therapeutic monoclonal antibodies, such as rituximab (anti-CD20) or trastuzumab (anti-HER2) by recognising their IgG component [39,44]. Researchers achieved cancer regression in vivo, first in CD20-positive B-lymphoblastoid cell lines using rituximab [39], and later in human HER2-positive BT474 breast cancer with trastuzumab engrafted in immunodeficient mice [40]. A later study, using anti-CD20, anti-HER2, and anti-GD2 antibodies to facilitate ADCC, along with a second generation CD16 CAR, demonstrated superior T cell activation, proliferation, and cytotoxicity compared to the first generation CD16 CAR [41]. Due to these promising results, the CD16 CAR is currently in clinical trials (NCT02776813, NCT03189836, NCT03266692, NCT03680560) for non-Hodgkin’s lymphoma, HER2-positive cancers, or multiple myeloma. However, while ADCC is a powerful mechanism in the control of some cancers, it has yet to be determined whether the downregulation of the NK cell receptor ligands from tumour cells will prevent complete clearance and long-term remission.

In another “lock-key” interaction, a biotin-binding immune receptor CAR (BBIR CAR), utilises biotin–avidin to act as an intermediary mechanism bridging the CAR T cell to an antigen [45] (Figure 2). By replacing a CAR scFv with an avidin molecule, it allows recognition of any biotinylated antigen-specific molecule from a number of sources and allows for multiple antigens to be targeted simultaneously based on diverse biotinylation. This approach has shown to result in both in vitro and in vivo tumour suppression [45]. This was replicated in a second study [46] with similar success, but concerns were raised regarding the immunogenicity of avidin, which caused off-target anti-tag effects. Therefore, whilst promising, this approach must be extensively tested in preclinical in vivo models to determine efficacy and safety.

Another approach is to use anti-tag CAR designs which utilise scFvs targeting molecular tags or chemically conjugated peptides which in turn bind to tumour antigens. Some tags that have been explored include the fluorescent label fluorescein isothiocyanate (FITC) [47,48,49] and a peptide neoepitope (PNE) [50,51,52]. This approach is quickly being adopted by a number of groups and are appealing as they introduce a measure of external control over the CAR [50]. This is best shown using a Nalm-6 xenograft model in which the switch anti-PNE CAR system was able to be dose-titrated by adjusting the concentration of antibody administered, with low antibody doses (0.5 mg/kg) maintaining a larger central memory CAR T cell population, improving long term CAR T cell persistence compared to high doses (2.5 mg/kg), which produced more effector CAR T cells [50].

Another example of a universal CAR using tag-specific interactions is the split, universal, programmable (SUPRA) CAR system. This design incorporates an extracellular leucine zipper as the CAR ectodomain, bound to the intracellular T cell signalling domains (zipCAR). The leucine zipper of the zipCAR binds a second leucine zipper on an scFv or other antigen-specific molecule (zipFv), allowing for T cell activation following zip–zip interaction toward any leucine-zipper bound molecule [53]. Using an immunodeficient NOD Scid gamma (NSG) xenograft mouse model, zipCAR T cells were shown to successfully eliminate HER2-positive SK-BR-3 human breast cancer cells grown subcutaneously following regular doses of anti-HER2 zipFv, with higher doses of the zipFv correlating with better killing but not affecting cytokine production [53]. Moreover, affinity tuning the interaction resulted in a correlation shown between increasing binding affinity and cytokine release [53].

Whilst these UIR platforms have yet to be tested in the clinic, their broad applicability and flexibility in various cancer types and antigen targets suggest that the UIRs are a promising strategy that allow for increased utility to be derived from the once off transduction of a patient’s T cells.

## 3. Designing the CAR: Selecting the Endodomain

Following antigen recognition by the CAR ectodomain, T cell activation is facilitated by the phosphorylation of the immunoreceptor tyrosine-based activation motifs (ITAMs) in the CD3ζ chain of the CAR endodomain. While the incorporation of a CD3ζ domain is sufficient to instigate T cell activation, the addition of costimulatory components into the CAR endodomain, such as CD28 or 4-1BB, significantly improves the activation, function, and persistence of the CAR T cells, as it provides the requisite secondary signals necessary for robust T cell activation [1,54].

There are a number of different costimulation molecules utilised in CAR T cell designs, including those from the CD28 family, such as CD28 [54] and inducible costimulatory (ICOS) [55,56], and those from the tumour necrosis factor receptor family (TNFR) consisting of CD27 [57], OX40 [58], and 4-1BB [22]. The two most popular costimulation molecules used clinically are CD28, which signals via a PI3K pathway inducing robust T cell proliferation and IL-2 production, and 4-1BB (CD137), further activating the nuclear factor (NF)-kB, mitogen-activated protein kinase (MAPK), and ERK signalling pathways [59,60]. In a recent study, a novel third-generation CD19 CAR integrating CD28 with the toll/interleukin-1 receptor (TIR) domain of toll-like receptor 2 (TLR2), a costimulatory receptor shown to enhance CAR T cell killing and expansion, was administered to three relapsed B-ALL patients, with all three patients achieving complete remission [61]. It is evident that the selection of a costimulation domain is critical to tailoring optimal CAR T cell function and fate, even if it remains unclear as to which domain is optimal for each receptor in each tumour type. Therefore, an increased focus on affinity-tuning the ectodomain and matching to the endodomain design will in time, enable more tailored T cell responses.

Recently, modulating the T cell signal strength by targeting the number of ITAMs within the CD3ζ signalling tail has also proved to be an effective way to tailor T cell signal strength and fine-tuning of CAR T cell potency [62]. This approach has recently been explored by Feucht and colleagues who explored the effect of ITAM number in the context of a CD28–CD3ζ CAR, demonstrating that a single proximal ITAM (1XX) was all that was required to maintain T cell function [63]. In this study, the CAR containing a single ITAM, proximal to the cell membrane (1XX), outperformed all other functional combinations of the three CD3ζ ITAMs in both in vitro and in vivo NALM6 mouse models of leukaemia [63]. Furthermore, these 1XX CAR T cells were less differentiated (proposed to be due to the intermediate levels of T cell signalling) compared to the fully phosphorylated and fully non-phosphorylated CAR T cells. This has been associated with increased anti-tumour function and persistence [64,65]. This study highlights the exquisite customization capable with all components of the CAR design and opens a number of possibilities for fine tuning of T cell signal strength.

The role of costimulation is best dissected when examining the influence on T cell signalling. A recent phosphoproteomic analysis comparing signalling via CD28/CD3ζ-CAR and a 4-1BB/CD3ζ-CAR independent of binding affinity showed that the inherent functional differences in these two CARs were due to kinetics and the magnitude of signal rather than the activation of separate pathways [66]. These data suggest that CAR T cell functional differences are due to signal intensity and strength rather than signalling via alternative pathways [66]. Interestingly, this study provided evidence for using the endodomain to influence signal strength in combination with affinity tuning the ectodomain. Historically, rapid and intense CD28/CD3ζ-CAR T cell phosphoprotein signalling has been associated with a more effector-like T cell phenotype with higher cytokine production and poorer CAR T cell persistence [67]. Recently, this approach to increase signal strength by altering the endodomain was investigated in a study focused on increasing the T cell signalling strength of low affinity (Kd 1.9 × 10^6^ mol/L) ectodomains [68]. In this combinatorial approach, functional responses via low affinity receptors could be enhanced by incorporation of a CD28 costimulation endodomain as compared to 4-1BB [68]. Our previous work has showed that affinity is associated with both rapidity of T cell cytotoxicity [69] and the time taken for T cell dissociation from the target cell [70]. Therefore, whilst the ectodomain is critical for antigen recognition and T cell activation, the endodomain selection will also be of critical importance in the overall design of effective receptors and tailoring of effective CAR T cell responses.

## 4. Multi-Targeted Approaches

Despite the increasing flexibility and variability possible with CAR design (for example using UIRs), there are still a number of obstacles affecting the successful translation of CAR T cell immunotherapy, particularly in the solid cancer setting. Despite impressive initial CD19 CAR T cell responses of up to 80% determined by 5-year overall survival, there is growing evidence of relapse due to antigen escape, with reports that 7–33% of responders in B-ALL relapse with CD19-negative tumours [35,71]. One of the strategies currently being employed to overcome this issue is to target multiple cancer antigens simultaneously, with the goal of eliminating heterogeneous tumours more rapidly. The targeting of multiple antigens can be done in several ways including: coadministration of CAR T cells with different specificities; simultaneous expression of CARs in T cells using a single vector (i.e., bispecific and tandem CARs, recently reviewed by Maizner and Mackall [72]); or encoding additional elements, such as bispecific T cell engagers (BiTEs) or cytokines to be secreted into the tumour microenvironment to recruit a robust diversified immune response.

Intra-tumour heterogeneity, especially in solid tumours, is a particular challenge for the field and was first demonstrated in 2014 through next generation sequencing, in which varying mutations (0 to 8000) were heterogeneously located throughout primary and metastatic glioblastoma [73]. At the protein level, EGFRvIII protein expression on cells has been shown to vary significantly depending on its location within a glioblastoma tumour [74,75]. Therefore, a broader multi-targeting approach would likely be necessary to address intra-tumoural heterogeneity.

### 4.1. Dual CAR

**Figure 3:**
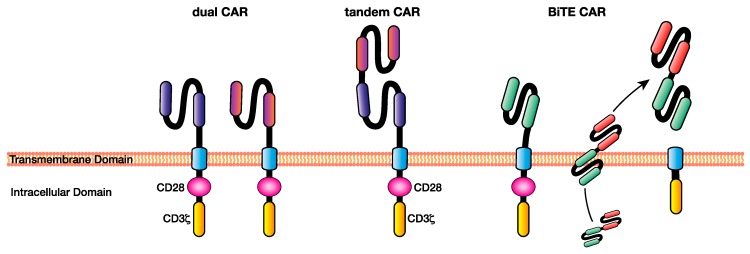
Multi-targeting chimeric antigen receptor (CAR) T cell approaches facilitate the elimination of heterogeneous cancers through the recognition of multiple antigens, overcoming some of the issues of single-targeting CAR T cell immunotherapy such as antigen escape. Examples of multi-antigen targeting CAR T-cell approaches include dual CARs, in which T cells express CARs specific for multiple antigens, tandem CARs, where two single-chain variable fragments (scFv’s) specific for different antigens are included in the ecto-domain separated by a Gly-Ser linker, and BiTE CARs, which are CAR T cells capable of secreting a BiTE targeting a different antigen to the CAR at the tumour site. BiTE: bi-specific T cell engager.

The dual CAR involves the simultaneous expression of CARs with different scFvs targeting two separate antigens. This is usually achieved by the inclusion of both CAR encoding sequences within the same bicistronic vector under the same promoter, enabling the simultaneous expression of both CARs at the T cell plasma membrane (Figure 3). It facilitates the simultaneous targeting of multiple tumour antigens that are differentially expressed within patient cancers [76,77].

Dual-specific CAR T cell therapies have been developed in response to the relapses following CD19 CAR T cell therapy. Earlier preclinical studies combined CD19 CARs with CARs targeting other B-cell specific markers such as CD22 [78], CD123 [77], and CD20 [79]. These strategies resulted in excellent elimination of B cell malignancy in mouse models above single-antigen targeted CAR T cells [77]. These data have resulted in phase I clinical trials of dual-specific CAR T cells targeting CD19 and CD22 (NCT03593109), and CD19 and CD20 (NCT03207178).

Dual CAR T cell therapies are also being developed in heterogeneous solid tumours such as glioblastoma using CAR T cells against HER2 and IL13Rα2 [76] and breast cancer targeting HER2 and MUC1 [80]. Ahmed and colleagues created a construct targeting three antigens and creating a trivalent CAR targeting HER2, IL3Rα2, and EphA2 for use in glioblastoma [81]. This study demonstrated that by using this single construct, a greater number of patients could be treated that express at least one of the target antigens, with the trivalent CAR therapy able to eliminate nearly all of the tumours cells in orthotopic patient-derived xenograft (PDX) mouse models [81].

### 4.2. TanCAR

In a variation to the dual CAR concept, the tandem CAR (or tanCAR) developed by Grada and colleagues involves the engineering two scFvs as an ectodomain, separated by a Gly-Ser linker, which enables simultaneous targeting of two antigens from a single receptor [82] (Figure 3).

The tanCAR involves additional complexity as the orientation and efficacy is influenced by the size and distribution of the two target antigens, therefore requiring extensive modelling [83]. In a study examining a bispecific tanCAR targeting CD19 and HER2, superior killing and cytokine release were demonstrated when both antigens were present on the target cell in vitro and in severe combined immune deficiency (SCID) mouse models of Daoy.TET.CD19 xenografts [82]. Further research by the same group suggests that tanCAR T cells targeting HER2 and IL13Rα2 simultaneously display greater cytotoxic killing and enhanced cytokine production compared to both dual CARs and pooled T cells expressing each single CAR receptor [83]. This superior anti-tumour action compared to other forms of bi-specificity was proposed to be due to their improved ability to simultaneously engage both antigens simultaneously through a single receptor, suggesting enhanced function is due to affinity/avidity differences in synapse formation. Interestingly, the tanCAR in this system did not appear to induce increased upregulation of activation markers programmed cell death protein 1 (PD1), lymphocyte activation gene 3 (LAG3), and T cell immunoglobulin and mucin domain-containing protein 3 (TIM3) over the separate dual-specific CAR T cells, suggesting comparable levels of T cell signalling and susceptibility to exhaustion between the tanCAR and dual CAR systems. Certainly, targeting tumour antigens simultaneously may provide an enhanced therapeutic approach to tackle antigen escape.

### 4.3. BiTE Secreting CAR T Cells

One of the advantages of a living drug such as CAR T cells is that they have been shown to infiltrate into dense tumours, cross the blood–brain barrier, and therefore have the potential to deliver drugs into the local tumour milieu. Therefore, CAR T cells can also be used to deliver drugs and soluble antibodies such as bispecific T cell engagers (BiTEs) into a solid tumour microenvironment [84]. BiTEs are bi-specific soluble antibodies targeting a tumour antigen on one arm and CD3ε on the other arm, resulting in an antibody that is capable of engaging T cell cytotoxicity of the tumour. They have shown some success in clinical trials [85]. While CAR T cells require ex vivo engineering to express tumour specific receptors, BiTEs can be used to engage bystander T cells to kill the tumour, which is an advantageous combination to enhance CAR T cell efficacy. This novel form of dual CAR targeting was demonstrated by Choi and colleagues to be effective in the clearance of heterogeneous EGFRvIII/EGFR expressing tumours in vivo, where a tumour-specific EGFRvIII CAR simultaneously secreted an EGFR-specific BiTE into the tumour microenvironment [84] (Figure 3). While the study was carried out in NSG mice, lacking an endogenous T cell population for the BiTE’s to engage, enhanced function was observed in vivo. Whether or not BiTE-secreting CAR T cells provide an advantage over CAR T cells alone by redirecting non-specific endogenous T cells to perform anti-tumour functions remains to be explored but offers an exciting new approach to tumour specific secretion of BiTEs or other anti-tumour biologicals.

## 5. Conclusions

With the clinical success of CAR T cell immunotherapy in the treatment of haematological cancers over the past decade, the field has rapidly expanded. However, these efforts have been less successful in a solid tumour context due to its highly immunosuppressive microenvironment and reduced CAR T cell persistence. Efficacious CAR T cell responses to heterogeneous and solid tumours will likely require novel technological advancements to the CAR ecto- and endo-domains to overcome the additional challenges of the tumour microenvironment. Although alterations can be made to CAR T cells to improve T cell co-stimulation and activation, they may also cause activation-induced cell death, highlighting the delicate balance necessary for an optimal CAR design. While CAR constructs are becoming more complex in order to meet the hostile environments of solid tumours and to ensure safety, there remains a lot more to learn, particularly regarding the underlying biology of CAR T cell therapy and how receptors fully integrate signal strength and function. Obstacles in the field such as antigen escape are being tackled innovatively through modifications to the base CAR design, such as the simultaneous multi-targeting of antigens by CAR T cells or UIRs. The modular nature of CAR design lends itself to innovative strategies in protein engineering, which will be essential to overcome the barriers presented by the tumour microenvironment. Recent years have seen significant progress in the field toward understanding how altering the ecto- and endo-domain of CAR impacts function and sets the groundwork for maximising CAR T cell responses in fighting cancer. By tailoring and integrating modifications to the ectodomain structure, such as through attuning the affinity of the binding interaction to the selection of optimal costimulation domains, we can start to tailor CAR T cell therapies to the full spectrum of cancer types.

## Abbreviations

CAR	Chimeric antigen receptor
dcCAR	Dual chain CAR
TRuC	T cell receptor fusion constructs
UIR	Universal immune receptors
tanCAR	Tandem chimeric antigen receptors
BiTE	Bi-specific T cell engagers
TCR	T cell receptor
MHC	Major histocompatibility complex
pMHC	Peptide major histocompatibility complex
B-ALL	Diffuse large B-cell lymphoma
scFv	Single chain variable fragment
TAA	Tumour-associated antigens
FSH	Follicle-stimulating hormone
NK cells	Natural killer cells
ADCC	Antibody-dependent cell cytotoxicity
BBIR	Biotin-binding immune receptor
FITC	Fluorescin isothiocyanate
PNE	Peptide neoepitope
SUPRA	Split, universal, programmable
ITAM	Immunoreceptor tyrosine-based activation motif
PDX	Patient-derived xenograft
